# Simulated basis sets for semi-LASER: the impact of including shaped RF pulses and magnetic field gradients

**DOI:** 10.1007/s10334-020-00900-1

**Published:** 2020-12-23

**Authors:** Oscar Jalnefjord, Patrick Pettersson, Lukas Lundholm, Maria Ljungberg

**Affiliations:** 1 Department of Radiation Physics, Institute of Clinical Sciences, Sahlgrenska Academy, University of Gothenburg, Gothenburg, Sweden; 2 Department of Medical Physics and Biomedical Engineering, MRI Center, Sahlgrenska University Hospital, Bruna stråket 13, 413 45 Gothenburg, Sweden

**Keywords:** Proton magnetic resonance spectroscopy (E05.196.867.519.775), Spectrum analysis( E05.196.867), Magnetic resonance imaging (E01.370.350.825.500)

## Abstract

**Objective:**

To study the need for inclusion of shaped RF pulses and magnetic field gradients in simulations of basis sets for the analysis of proton MR spectra of single voxels of the brain acquired with a semi-LASER pulse sequence.

**Materials and methods:**

MRS basis sets where simulated at different echo times with hard RF pulses as well as with shaped RF pulses without or with magnetic field gradients included. The influence on metabolite concentration quantification was assessed using both phantom and in vivo measurements. For comparison, simulations and measurements were performed with the PRESS pulse sequence.

**Results:**

The effect of including gradients in the simulations was smaller for semi-LASER than for PRESS, however, still noticeable. The difference was larger for strongly coupled metabolites and at longer echo times. Metabolite quantification using semi-LASER was thereby less dependent on the inclusion of gradients than PRESS, which was seen in both phantom and in vivo measurements.

**Discussion:**

The inclusion of the shaped RF pulses and magnetic field gradients in the simulation of basis sets for semi-LASER is only important for strongly coupled metabolites. If computational time is a limiting factor, simple simulations with hard RF pulses can provide almost as accurate metabolite quantification as those that include the chemical-shift related displacement.

**Supplementary Information:**

The online version contains supplementary material available at 10.1007/s10334-020-00900-1.

## Introduction

Magnetic resonance spectroscopy (MRS) is a unique technique in the sense that it can provide insight into the cell metabolism completely non-invasively. It can be used in a broad range of applications e.g. to detect and stage brain tumors [1], determine tumor treatment response [2], and to study neurodegeneration [3] and psychiatric disorders [4]. However, although MR spectroscopy has been available since the advent of clinical MR, it has not yet become a widespread clinical tool mainly since both data acquisition and analysis requires specially trained personnel. The acquisition methods and their implementation have also varied between vendors and hospitals, making it hard to compare results [5].

High magnetic field strength, 3 T for the clinical setting, is beneficial for MRS as it provides a stronger signal and a wider separation of chemical shifts of resonances in absolute numbers, thus enabling separation of a larger number of metabolites [6]. However, for the pulse sequences typically used clinically, e.g. PRESS, the bandwidth of the RF pulses is relatively low [7]. The larger chemical shift separation, therefore, also results in a more problematic localization of the volume-of-interest (VOI) since its position in space depends on the resonance frequencies [8]. Correspondingly, the different peaks in a spectrum will originate from slightly different regions in space. An even more problematic situation occurs for strongly coupled metabolites where only a subset of the spins in a metabolite experience the intended slice selective RF pulses, thus giving rise to unexpected or absent J-coupling effects and potentially signal loss [8, 9]. Consequently, the obtained spectra can differ substantially from what would be expected without the localization errors both in terms of quality and appearance.

To mitigate the problems related to localization errors with e.g. PRESS, it has been suggested to use adiabatic RF pulses, especially for the refocusing pulses which typically have the lowest bandwidth and thereby give rise to the largest localization errors [7]. The most clinically relevant implementation is the semi-LASER pulse sequence, which can achieve echo times almost as short as PRESS, but with a substantially reduced localization error [10, 11].

Analysis of MRS data typically revolves around translating an MR spectrum into (relative) tissue concentrations of different metabolites. In simplistic cases with well-separated metabolite resonances it may be possible to obtain these concentrations simply by calculating the area under a peak of interest in a given interval [12]. However, in more realistic cases of coupled and overlapping resonances, prior information must be incorporated into the analysis. One way of doing this is to obtain a set of high-quality spectra for each metabolite separately to be used as a basis for the subsequent analysis [12]. Basis sets have historically been acquired experimentally where metabolite-specific phantoms were produced and measurements with the exact pulse sequence parameters were made. Due to the difficulty in producing high-quality phantoms, the time-consuming task of acquiring spectra for each metabolite and pulse sequence of interest as well as the improvement of hardware stability during the last two decades, a transition towards the use of simulated basis sets has been seen [13–18]. After an initial high workload when setting up the simulation framework, very little manpower is needed to generate new basis sets for other pulse sequence timings, metabolites or even pulse sequences. Different levels of detail of the simulations can be chosen; in the simplest setting hard RF pulses are simulated, a next step is to use the actual shaped RF pulses and in the most realistic and computationally demanding approach magnet field gradients are also incorporated. For the simulation of basis sets for the PRESS pulse sequence, it has previously been shown to be critical to include the gradient effects for strongly coupled metabolites to properly account for localization errors [19]. However, it is not obvious if this applies to semi-LASER for which the localization errors are smaller.

In a recent consensus paper, it was pointed out that the use of semi-LASER and simulated basis sets are two of the three most important factors for successful future use of MRS in the clinical setting [5]. Most published studies related to the evaluation of simulated basis sets have focused their analysis on PRESS and the nowadays less commonly used pulse sequence STEAM, and to our knowledge only one study has explicitly studied simulated basis sets for semi-LASER, however, also this study focuses primarily on PRESS and STEAM, and to some extent the MEGA version of semi-LASER [18].

Since little work has been published related to the specific topic of simulated basis sets for the semi-LASER pulse sequence the aim of this study was to analyze simulation aspects for the generation of high-quality basis sets for semi-LASER. Given that the use of a potentially too simplistic simulation of basis sets can be expected to mainly introduce a systematic error, i.e. a deterministic discrepancy between the basis set and acquired data, the study was designed to have a particular focus on the accuracy of the spectral analysis.

## Materials and methods

To study the effect of basis-set simulation complexity for the semi-LASER pulse sequence, basis sets were generated and compared among different complexity levels and echo times. Phantom and in vivo measurements were performed to evaluate the impact on metabolite quantification. For comparison, all simulations and measurements were also performed with the PRESS pulse sequence.

### Phantom and in vivo measurements

The phantom measurements were performed using the MRS Braino phantom (General Electric Medical Systems, Milwaukee, WI, USA), which contains six of the most relevant brain metabolites: N-acetylaspartate (NAA; 12.5 mM), glutamate (Glu; 12.5 mM), creatine (Cr; 10 mM), myo-inositol (mI; 7.5 mM), lactate (Lac; 5 mM) and choline (Cho; 3 mM). The phantom also contained the chemicals sodium azide (0.1%), potassium phosphate monobasic (KH_2_PO_4_; 50 mM), sodium hydroxide (NaOH; 56 mM), and Gd-DPTA (Magnevist; 1 ml/l). All phantom MRS measurements were performed at 23 degrees Celsius (room temperature). The volume of interest (VOI) was positioned in the center of the phantom.

In vivo measurements were performed on one healthy volunteer (male, 24 years), who provided informed consent. The in vivo measurement was performed as a part of a larger study that was approved by the Swedish Ethical Review Authority. The VOI was positioned in centrum semiovale (see VOI position in Supplementary Fig. S1).

MRS data were acquired on a Philips Ingenia 3 T MR system (software release 5.7), equipped with a 32-channel head coil and a Omega HP gradient system using mode 2 in the spectroscopy measurements (45 mT/m and 120 T/m/s). Data were acquired with the semi-LASER pulse sequence at echo times 35, 45, 55, 65, 75 and 85 ms.

Acquisition parameters common to both the phantom and in vivo measurements were: the size of the excited volume: 20 × 20 × 20 mm^3^, receiver bandwidth: 2000 Hz, NSA: 128, water suppression: VAPOR with a window width of 90 Hz [20]. To enable a subsequent quantitative analysis, non-water-suppressed spectra were also acquired with the same scan parameters (NSA: 16). The semi-LASER pulse sequence consisted of an excitation RF pulse with flip angle 90 degrees and bandwidth (full width at half maximum) 3238 Hz, and two pairs of adiabatic refocusing RF pulses with flip angle 180 degrees and bandwidth 5614 Hz, all RF pulses with a max B1 of 22.0 µT. For comparison, corresponding data was acquired with the PRESS pulse sequence using the same acquisition parameters. The PRESS pulse sequence consisted of an excitation RF pulse with flip angle 90 degrees and bandwidth 1987 Hz, and two refocusing RF pulses with flip angle 180 degrees and bandwidth 1267 Hz, all RF pulses with a max B1 of 13.5 µT. The shape of the excitation RF pulse was the same for both pulse sequences (asymmetric sinc), while the refocusing pulses were offset independent trapezoid pulses for semi-LASER and a numerically optimized, amplitude-modulated pulse for PRESS [21].

Specific acquisition parameters for the phantom measurements were TR: 5000 ms, acquired samples: 4096, phase cycles: 16 (semi-LASER and PRESS). Corresponding parameters for the in vivo measurements were TR: 2000 ms, acquired samples: 1024 and phase cycles: 32 (semi-LASER) and 16 (PRESS). Semi-LASER used a basic nested phased cycling scheme with a nesting order of 2nd, 3rd, 4th refocusing pulse followed by the excitation pulse in the innermost loop. All pulses used a 180º increment except for the 4th refocusing pulse which was incremented by 90º. PRESS used an EXORCYCLE phase cycling scheme.

### Simulation of basis sets

Basis sets for analysis of the MRS data were generated through numerical calculations based on the quantum mechanical density-matrix formalism [22] following the algorithm outlined by Zhang et al. [16]. For each time interval with a constant Hamiltonian, i.e. constant RF and gradient fields, the time evolution of the spin-density operator is given by:

1 $$\rho_{n + 1} = U_{n} \rho_{n} U_{n}^{\dag }$$

where ${\rho }_{n}$ is the spin-density operator at the start of the *n*th time interval, ${U}_{n}$ is the propagator of the *n*th time interval and $\dag$ denotes the Hermitian conjugate. The starting point ${\rho }_{0}$ is given by the thermal equilibrium. The propagator ${U}_{n}$ is calculated as:

2 $${U}_{n}=\mathrm{exp}\{-i{H}_{n}\left({t}_{n+1}-{t}_{n}\right)\}$$

where exp denotes matrix exponential, ${H}_{n}$ is the Hamiltonian operator in the rotating frame of reference in the *n*th time interval, and ${t}_{n}$ and ${t}_{n+1}$ are the starting time points of the *n*th and (*n* + 1)th time intervals, respectively. The Hamiltonian operator is given by:

3 $${H}_{n}=\sum_{j}{\Omega }_{j}{I}_{jz}+2\pi \sum_{j,k}{J}_{jk}{{\varvec{I}}}_{j}\cdot {{\varvec{I}}}_{k}+\gamma {{\varvec{G}}}_{n}\cdot {\varvec{r}}\sum_{j}{I}_{jz}+\left[{\omega }_{n}^{nut}\left({{\varvec{I}}}_{x}\mathrm{cos}{\phi }_{n}+{{\varvec{I}}}_{y}\mathrm{sin}{\phi }_{n}\right)+{\Omega }_{n}^{rf}{{\varvec{I}}}_{z}\right]$$

where ${\Omega }_{j}$ is the chemical shift of spin $j$ in the rotating frame of reference relative to the chosen reference frequency, ${J}_{jk}$ is the J-coupling constant (in Hz) between spins $j$ and $k$, ${{\varvec{G}}}_{n}$ is the gradient vector during the *n*th time interval, ${\varvec{r}}$ is the position vector, while ${\omega }_{n}^{nut}$, ${\phi }_{n}$ and ${\Omega }_{n}^{rf}$ are the nutation frequency, phase and frequency offset of the RF pulse during the *n*th time interval, respectively. ${{\varvec{I}}}_{x}$,${{\varvec{I}}}_{y}$, ${{\varvec{I}}}_{z}$ are angular momentum operators which are conveniently expressed in matrix form [22]. Note that the two last terms in Eq. 3, relating to the effects of gradients and RF pulses, are only non-zero in cases when gradients or RF pulses are applied.

The chemical shifts and J-coupling constants needed to evaluate Eq. 3 were obtained from the literature [23, 24]. A linear model was applied to compensate for the fact that the literature values for chemical shifts and J-coupling constants were measured at human body temperature, while the phantom measurements in the current study were performed at room temperature [25].

Three levels of simulation complexity were considered: (1) hard RF pulses, (2) shaped RF pulses without gradients applied, and (3) shaped RF pulses with gradients applied. The first and second methods are associated with shorter computational time and the first also removes the need of knowledge of RF pulse shapes, but only the third one can capture the effects of varying chemical shifts on spatial localization. To incorporate the effects of gradients, simulations were performed on a grid of points in space and the final result was then given by summation over the grid. The geometric size of the grid was set to a cube with side 4.0 cm (twice the measurement volume side), while the number of grid points was set to 200 in each dimension (empirically found to be sufficiently large to make the geometrical discretization error negligible; data not shown). However, direct simulation on such a large grid is associated with a long computational time since the time is proportional to the product of the number of grid points in each dimension. To improve the computational speed, the one-dimensional projection method was used, which instead gives a computational time proportional to the sum of the number of grid points in each dimensions [16]. For the simulations without gradients, i.e. the first and second methods, the grid was only a single point in the origin of the coordinate system. In the first method rectangular RF pulses with duration 1 µs was used with spacing given by the distance between the magnetic centers of the shaped RF pulses. Pulse-sequence data, including RF and gradient pulse shapes and timing was extracted from the scanner software using custom MATLAB code.

MATLAB 2018 (MathWorks, Natick, MA, USA) was used for all simulations. The computational time per TE and pulse sequence was approximately 5 s, 1.5 min and 4 h for simulations with the hard RF pulses, shaped RF pulses without gradients applied, and shaped RF pulses with gradients applied, respectively, using a laptop with 3.3 GHz quadcore processor and 8 GB RAM.

### Data analysis

Spectral fitting was performed using LCModel [26] with the custom-made basis sets described above. The basis sets used to analyze the phantom data included the metabolites contained in the phantom, while the basis sets used to analyze the in vivo data additionally included phosphocholine (PCh), glycerophosphocholine (GPC), phosphocreatine (PCr), glycine (Gly), N-acetylaspartylglutamate (NAAG), glutamine (Gln), glutathione (Glt), gamma-aminobutyric acid (GABA), aspartate (Asp) and guanine (Gua), but excluded lactate. The reported values from the in vivo analysis are Cho + PCh + GPC, NAA + NAAG, Ins + Gly, Cr + PCr and Glu + Gln. For the analysis of phantom data, the LCModel control parameter *nobase* was set to true to ensure that any systematic differences between measured data and basis sets transitioned into the residual of the fit. For each TE, the control parameter *atth2o* was calculated based on the water T2 estimated from the non-water-suppressed measurements. The T2 estimation was performed in two steps, first the signal strength at each TE was estimated as the model intercept of a monoexponential fit to the FID, excluding the first five datapoints. Second, the signal strengths from all TEs obtained from the first step, were used to fit another monoexponential model where the slope was used as the estimate of the T2 of water in the specific experiment. This two-step approach minimizes the influence of artificial signal contributions in the beginning of the FID in each individual measurement.

To obtain estimates of metabolite concentrations without influence of transverse relaxation, an exponential model was fitted to the TE-dependent results from the LCModel analysis for each basis set. The model intercept at TE = 0 ms was used as the final estimate of metabolite concentration.

All data analysis was performed using Python 3.6 with matplotlib for visualization and scipy for exponential fitting. Numpy and pandas were used for numerical calculations and data handling, respectively.

## Results

### Basis sets

**Fig. 1:**
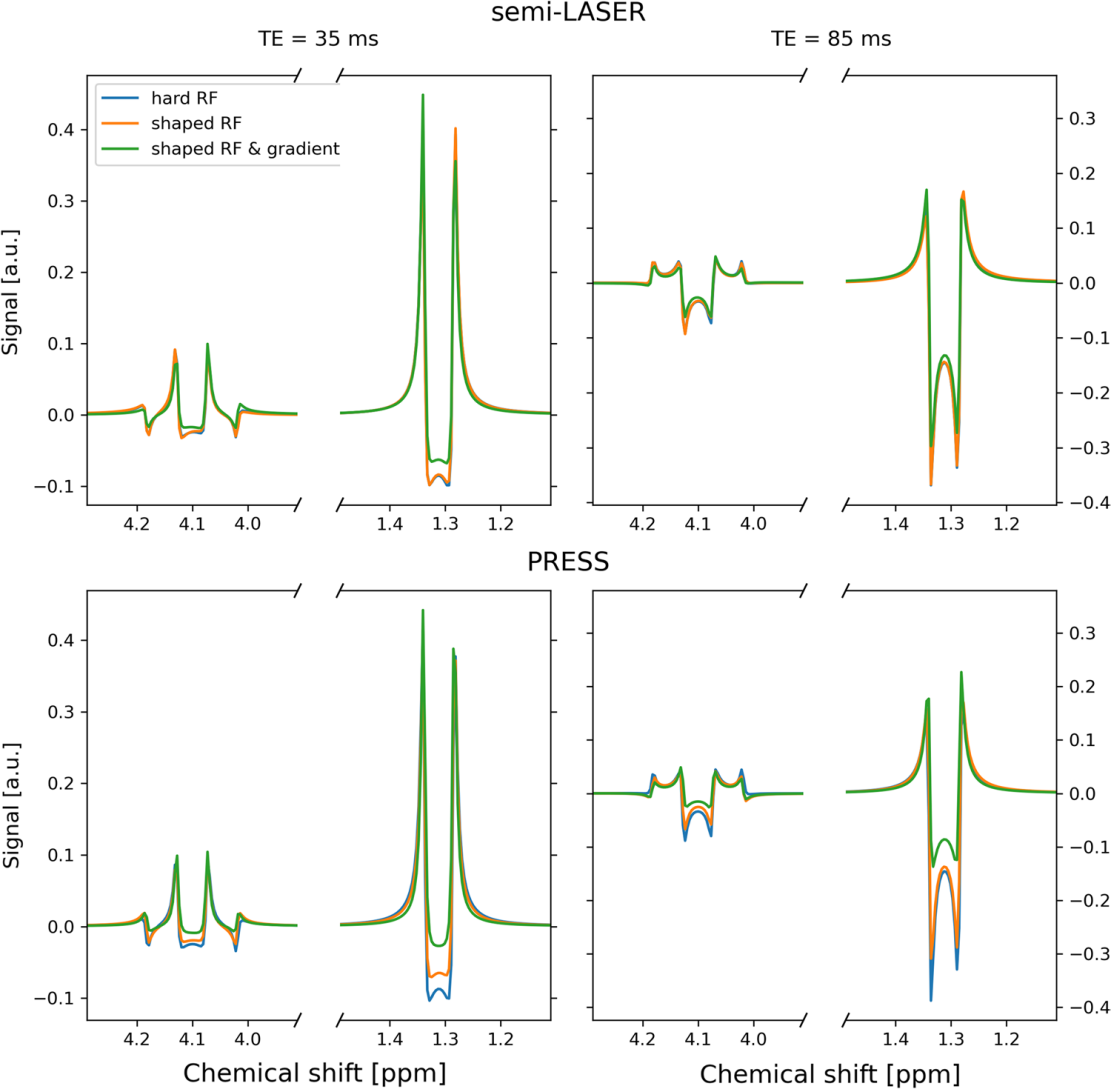
Simulated spectra of lactate at TE = 35 and 85 ms, for different simulation methods and pulses sequences. Note the broken x-axis that is used for improved visualization. See Supporting Figures S2–S7 for a complete set of TEs and metabolites

Inclusion of gradients in the simulations had an evident effect on the generated basis sets. Lactate, the most strongly coupled metabolite, is shown for two TEs in Fig. 1. Spectra for each metabolite and TE for both pulse sequences are shown in Supplementary Figures S2–S7. As an expected consequence of the higher RF bandwidth and correspondingly smaller localization artefacts, the effect of including gradients in the simulations was smaller for semi-LASER than for PRESS, but still noticeable, as seen for example at 1.3 ppm and TE = 35 ms for lactate (Fig. 1). The difference between hard RF pulses and shaped RF pulses in the simulations did not result in visually different results for semi-LASER, however, for PRESS there is a difference for e.g. lactate.

**Fig. 2:**
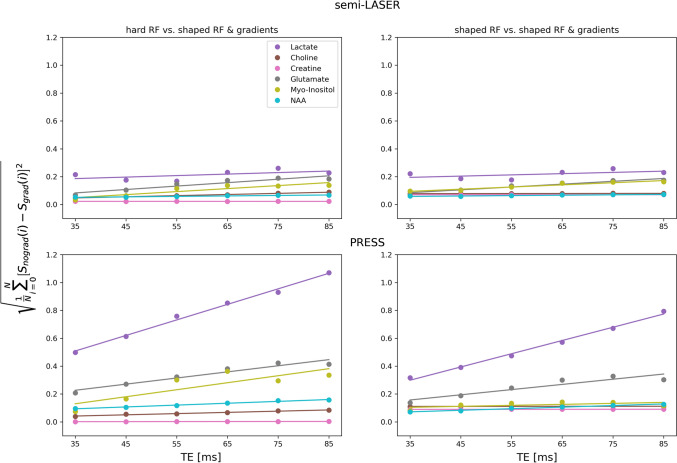
Square root of mean relative squared difference between spectra from simulations including gradients and other simulation methods for each TE and metabolite. This figure summarizes the differences displayed in Supporting Figures S2–S7

As seen in Fig. 2, the effect of including gradients in the simulations was more pronounced for more strongly coupled metabolites, which is due to the origin of the localization artefact. For semi-LASER, this effect was relatively independent of the TE, while for PRESS the effect increased with increasing TE. The difference between the use of hard and shaped RF pulses was relatively small but somewhat more pronounced for PRESS.

### Phantom data

**Fig. 3:**
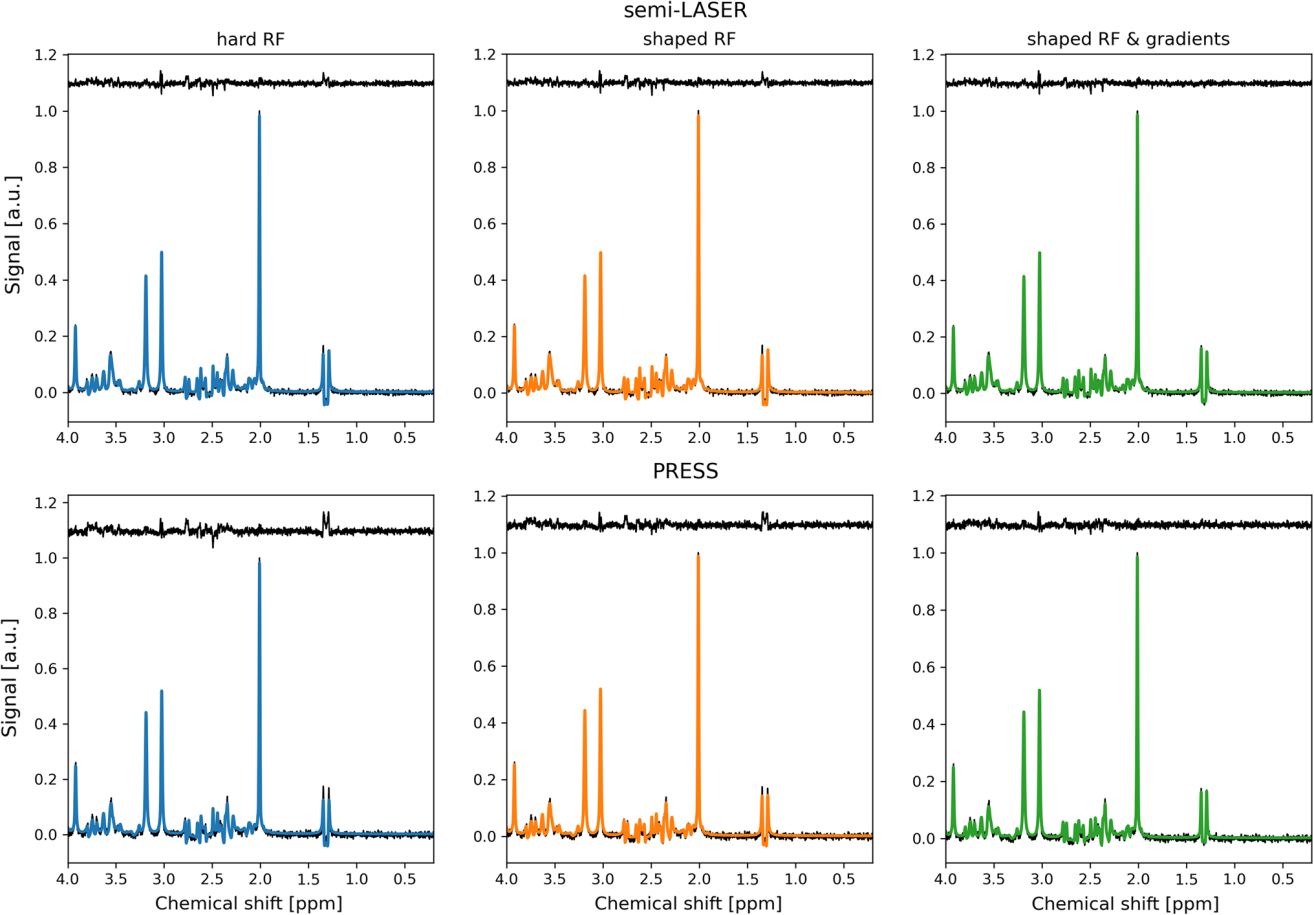
Measured phantom spectrum (black), LCModel fit (colored) and residual (black elevated) at TE = 35 ms for each simulation method and pulse sequence. The amplitude of the spectra was normalized relative to the NAA peak and an offset equal to 1.1 was added to the residual for improved visualization

The simulated basis sets did in general fit well to the data, but some structured residuals were observed (Fig. 3 and Supplementary Figs S8–S12). These structured residuals were seen at 1.3–1.4 ppm (lactate), 2.2–2.8 ppm (glutamate and NAA) and 3.0 ppm (creatine). The residuals in the lactate region were smaller but still noticeable for semi-LASER, compared with PRESS when gradients were not included in the simulations. Including the effects of gradients removed all structured residuals in the lactate region for both semi-LASER and PRESS. The residuals in the glutamate and NAA region followed a similar trend with smaller residuals for semi-LASER with the difference that at a close inspection some remaining structured residual could be seen even when the effects of gradients were simulated. The residuals in the creatine region were mainly unaffected by simulation methodology.

**Fig. 4:**
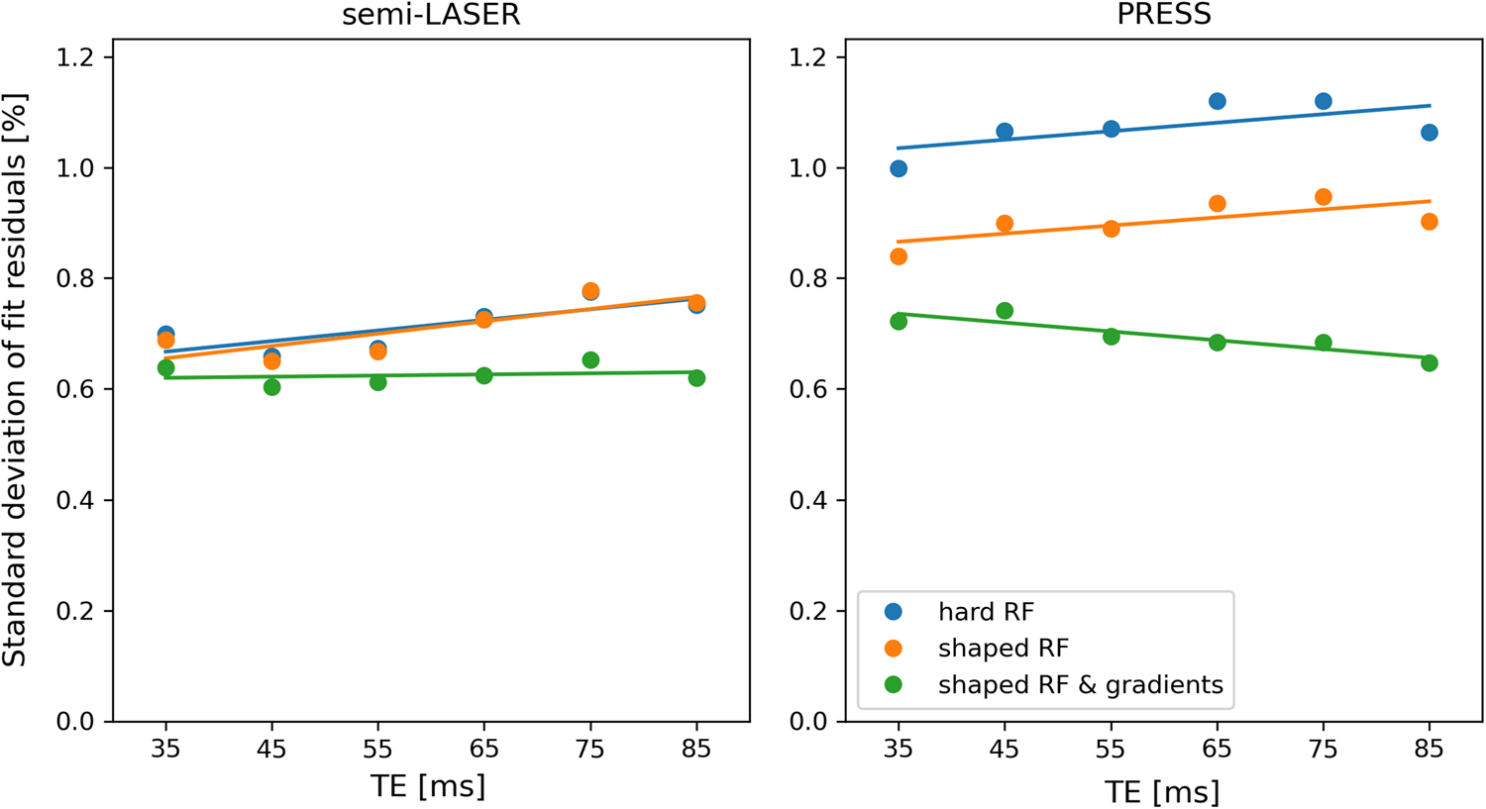
Standard deviation of LCModel residual at different TEs for each simulation method when analyzing the phantom data. The residual is given relative to the amplitude of the NAA peak as in Fig. 3 but also corrected for the T2 relaxation of NAA. Linear fits to the data are superimposed to show the trends in data

The size of the structured residuals tended to increase with increasing TE when the effect of gradients was not included in the simulations (Fig. 4). This is in agreement with Fig. 2, indicating that the origin of the structured residual is mainly related to chemical-shift related displacement errors.

semi-LASER showed in most cases a better ability for absolute quantification compared with PRESS when using basis sets based on simulations not including the effects of gradients (Supplementary Fig. 13 shows a comparison between concentrations extrapolated to TE = 0 ms and those prescribed by the vendor). The main differences were seen for the coupled metabolites lactate and myo-inositol. When the effects of gradients were included in the simulations, the performance of the two pulse sequences was similar. However, it should be noted that the monoexponential model used to extrapolate to TE = 0 ms did not fit very well to the data for some metabolites, in particular glutamate and myo-inositol (Supplementary Fig. S14).

### In vivo data

**Fig. 5:**
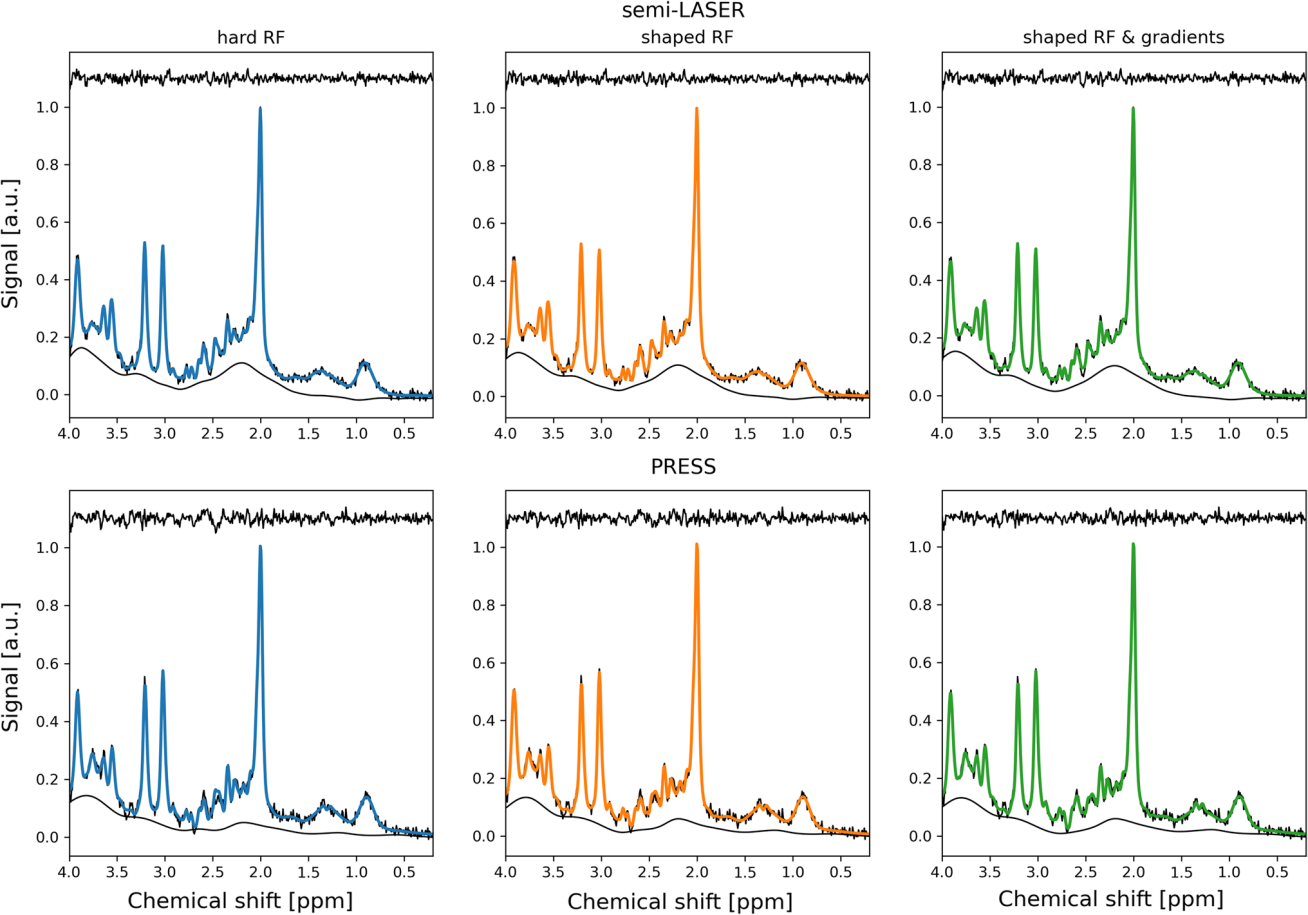
Measured in vivo spectrum (black), LCModel fit (colored) and residual (black elevated) at TE = 35 ms for each simulation method and pulse sequence. As in Fig. 3, the amplitude of the spectra was normalized relative to the NAA peak and an offset equal to 1.1 was added to the residual for improved visualization

Overall, the same trends as for the phantom measurements were found in vivo. The simulated basis sets did in general fit well to the in vivo data, however, also here some structured residuals were observed, in particular when gradients were not included in the simulations (Fig. 5 and Supplementary Figs S15–S19).

**Fig. 6:**
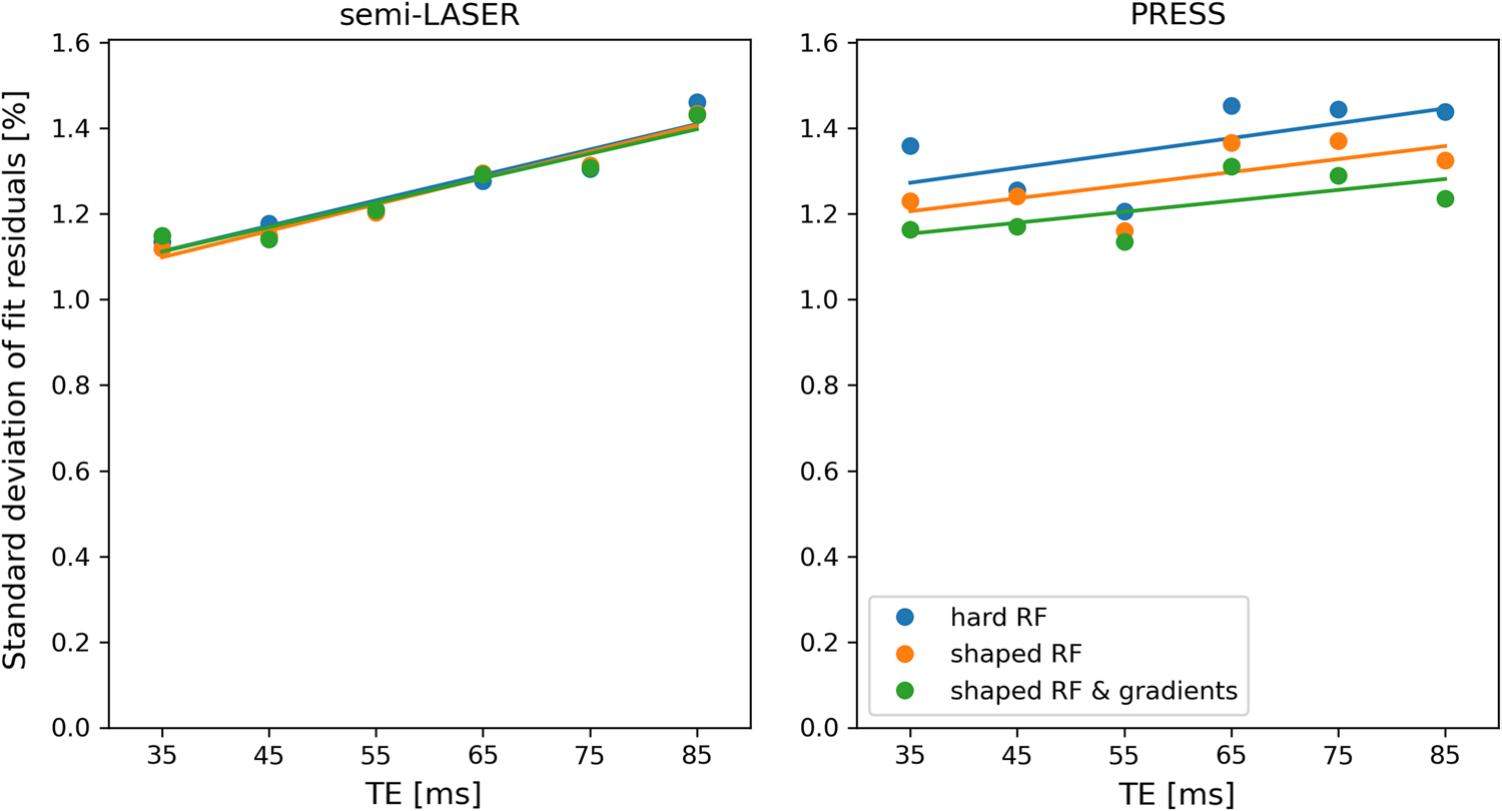
Standard deviation of LCModel residual at different TEs for each simulation method when analyzing the in vivo data similar to Fig. 4. The residual is given relative to the amplitude of the NAA peak as in Fig. 5 but also corrected for the T2 relaxation of NAA. Linear fits to the data are superimposed to show the trends in data

The standard deviation of the structured residuals was similar irrespective of the complexity of the basis set simulations for semi-LASER, while it increased with decreasing complexity for PRESS (Fig. 6). The residuals for PRESS simulated with shaped RF pulses and gradients were similar to the ones obtained with semi-LASER, irrespective of basis sets. There was a tendency for the standard deviation to increase with increasing TE for all three versions of simulated basis sets and both pulse sequences.

Semi-LASER showed less dependence than PRESS on the chosen simulation method regarding estimated concentrations in vivo (concentration estimates extrapolated to TE = 0 ms are shown in Supplementary Fig. S20), which is in agreement with the phantom measurements. The estimated concentrations for Cho, Cr and Ins was similar for PRESS and semi-LASER, however, for Glx and NAA PRESS estimated higher concentrations than semi-LASER.

As for the phantom measurements, the monoexponential model used to extrapolate to TE = 0 ms, did not fit very well to the data for some metabolites, in particular glutamate and myo-inositol (Supplementary Fig. S21).

## Discussion

Using the pulse sequence semi-LASER and simulated basis sets for analysis were pointed out as two of the three most important points for successful clinical application of proton MR spectroscopy of the brain at 3 T [5]. Yet, little work has been presented regarding simulating basis sets for semi-LASER. In this study, the need for computationally demanding simulations including shaped RF pulses and magnetic field gradients was evaluated through simulations as well as phantom and in vivo measurements. The results show that simple simulations using hard RF pulses are sufficient for most brain metabolites. However, for strongly coupled metabolites, such as lactate, it was beneficial to perform the more complex simulations. This is in stark contrast to the PRESS pulse sequence, where it was highly beneficial with a proper simulation of the chemical-shift related displacement for most metabolites.

The estimated phantom metabolite concentrations were close to those given by the phantom vendor for most metabolites. For myo-inositol and glutamate, the TE dependent concentration estimates did not follow a simple monoexponential model, possibly an indication of the inherent difficulty associated with analyzing spectra with complicated and overlapping metabolite spectra. Another explanation could be the fact that the complicated spectral modulation introduced by the J couplings will give time-dependent biases with potentially varying sign and magnitude. The in vivo concentration estimates followed trends very similar to those from the phantom and were in line with previously reported concentrations in healthy white matter [27].

Among the studied metabolites, lactate, the most strongly coupled metabolite, was clearly the one where inclusion of shaped RF pulses and magnetic field gradient in the simulations had the greatest impact as seen in Fig. 2. Similar, but smaller effects could be seen for the other coupled metabolites, while the uncoupled metabolites did not depend on simulation complexity. When comparing Figs. 4 and 6, it is evident that the different residual sizes for different simulation approaches for semi-LASER are only seen in the phantom data. Knowing that lactate is below detection level in healthy tissue and relating Fig. 2 to Figs. 4 and 6, make it clear that this difference between the phantom and in vivo results is due to the different concentrations of lactate, while other coupled metabolites produced negligible structured residuals in the semi-LASER analysis. This is in contrast to PRESS where distinctly different residual sizes could be seen also in the in vivo results, indicating that also the other coupled metabolites produce non-negligible structured residuals, as also can be seen in the individual spectra.

Although the results show that it is possible to achieve LCModel fits to PRESS data with very small residuals and limited metabolite concentration bias if true RF-pulse shapes and gradients are incorporated in the simulation of basis sets, it is worth noting that it may still be beneficial to use semi-LASER for in vivo MRS. The main reason is that most lesions are small, and to minimize signal from normal tissue the volumetric shift should be as small as possible. It is also more straightforward to interpret the results if the signal from all metabolites are acquired from the same region in space, since there otherwise may be different proportions of normal to pathological tissue for different metabolites. Knowing that the signal of all metabolites originate from the same region in space can be used to increase the size of the VOI, thus increasing the SNR of the measurement, since one does not have to take the VOI displacement error into account while planning the position of the VOI.

While the inclusion of shaped RF pulses and magnetic field gradients in the simulation of basis sets for semi-LASER provide superior results if strongly coupled metabolites are to be analyzed, the simple simulations with hard RF pulses is able to produce fairly accurate spectra even for these metabolites. This can be useful for example in pulse sequence optimization where computational complexity may be a limiting factor.

The results of the current study are based on a cubic VOI with a side of 2 cm. This is a size typical for clinical MRS that provides a compromise between SNR and localization specificity within a reasonable examination time. However, the results can be extended to any VOI size as long as only the gradient strength is used for changing the volume, i.e. the bandwidths of the RF pulses are kept constant, as in the actual implementation used in the current study. Under this assumption, the results can even be extended to the larger VOI sizes used in MR Spectroscopic imaging (CSI or MRSI), although different effects will be seen for MRSI since the signal is obtained from different voxels rather than from the entire VOI. The chemical shift displacement artifact is, for example, only evident in the voxels close to the border of the VOI, while voxels in the center of the VOI is free from this artifact due to the spatial encoding.

There are currently several versions of semi-LASER available, where the Philips implementation which was used in this study is one. While some results may depend on the particular implementation, the general results should be applicable to other similar implementations as well, which is important for example in the case of a future consensus implementation [28].

In conclusion, the inclusion of the shaped RF pulses and magnetic field gradients in the simulation of basis sets for semi-LASER is only important for strongly coupled metabolites. If computational time is a limiting factor, simple simulations with hard RF pulses can provide almost as accurate metabolite quantification as those that include the chemical-shift related displacement. This is in contrast to PRESS where a substantial improvement in metabolite quantification accuracy could be seen when including shaped RF pulses and magnetic field gradients in the simulation.

## Supplementary Information

Below is the link to the electronic supplementary material.

Supplementary file1 Supplementary figures: Fig S1 VOI position used for in vivo measurements. Figs S2-S7 as fig 1 but for all metabolites and TEs. Figs S8-S12 as fig 3 but for remaining TEs. Fig S13 Estimated phantom metabolite concentrations corrected for T2 relaxation (extrapolated to TE = 0 ms based on monoexponential fit of multi-TE data) and bias relative to specified concentrations. Fig S14 estimated phantom metabolite concentrations vs TE and exponential model fit (related to Fig 6). Figs S15-S19 as Fig 5 but for remaining TEs. Fig S20 Estimated in vivo metabolite concentrations corrected for T2 relaxation (extrapolated to TE = 0 ms based on monoexponential fit of multi-TE data). Fig S21 Estimated in vivo metabolite concentrations vs TE and exponential model fit (related to Supporting Fig S20) (PDF 2032 KB)
