# Supplementary material for: Simulated basis sets for semi-LASER: the impact of including shaped RF pulses and magnetic field gradients
Source: MAGMA. 2020 Dec 23;34(4):545–54. doi: 10.1007/s10334-020-00900-1 (PMC8338815; doi:10.1007/s10334-020-00900-1)

Supplementary material

Figure S1. VOI position used for in vivo measurements shown on a FLAIR image acquired for VOI planning

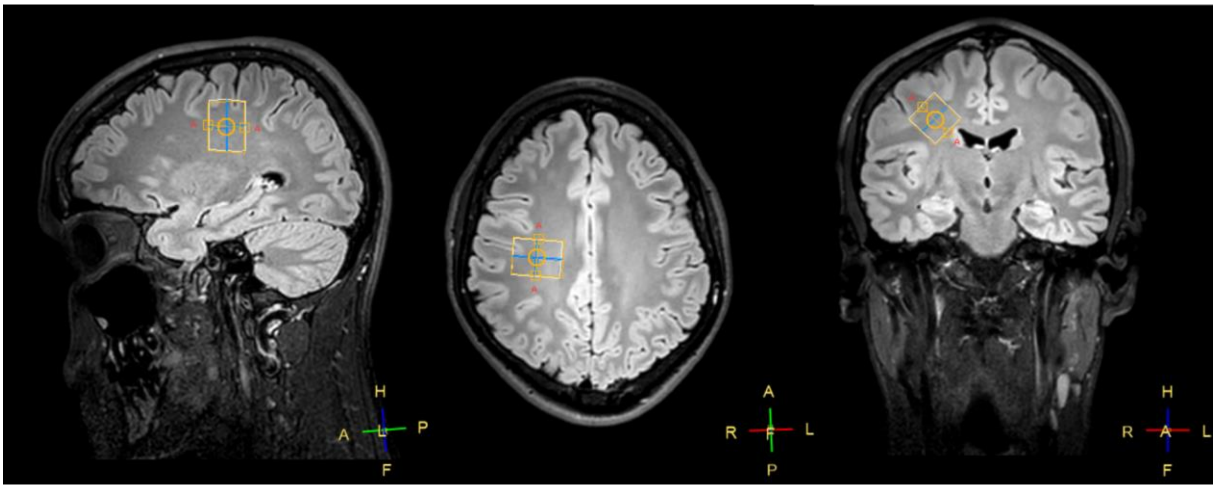

Supplementary figures S2-S7. Simulated spectra for different simulation methods and pulse sequences.

S2. Lactate

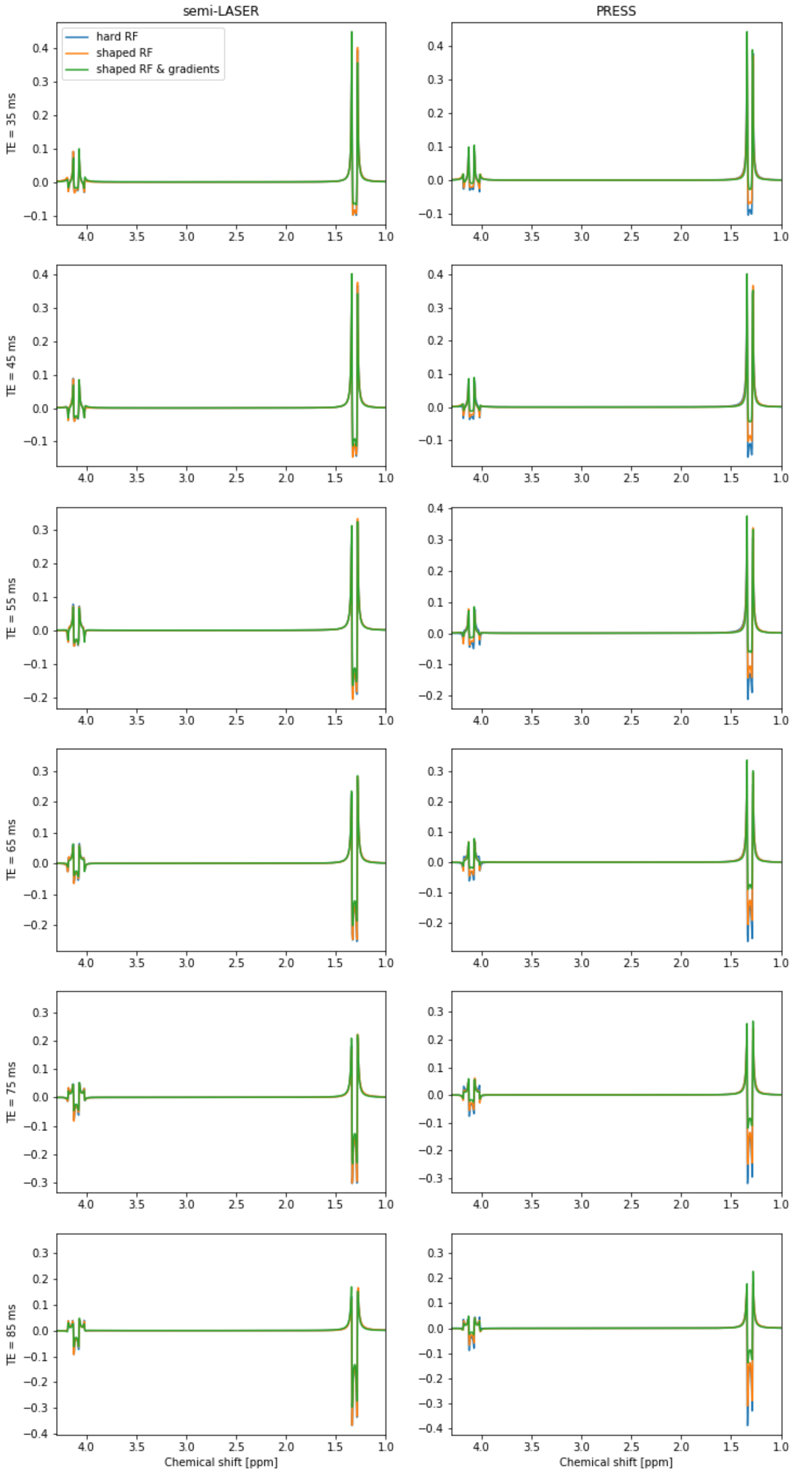

S3. Choline

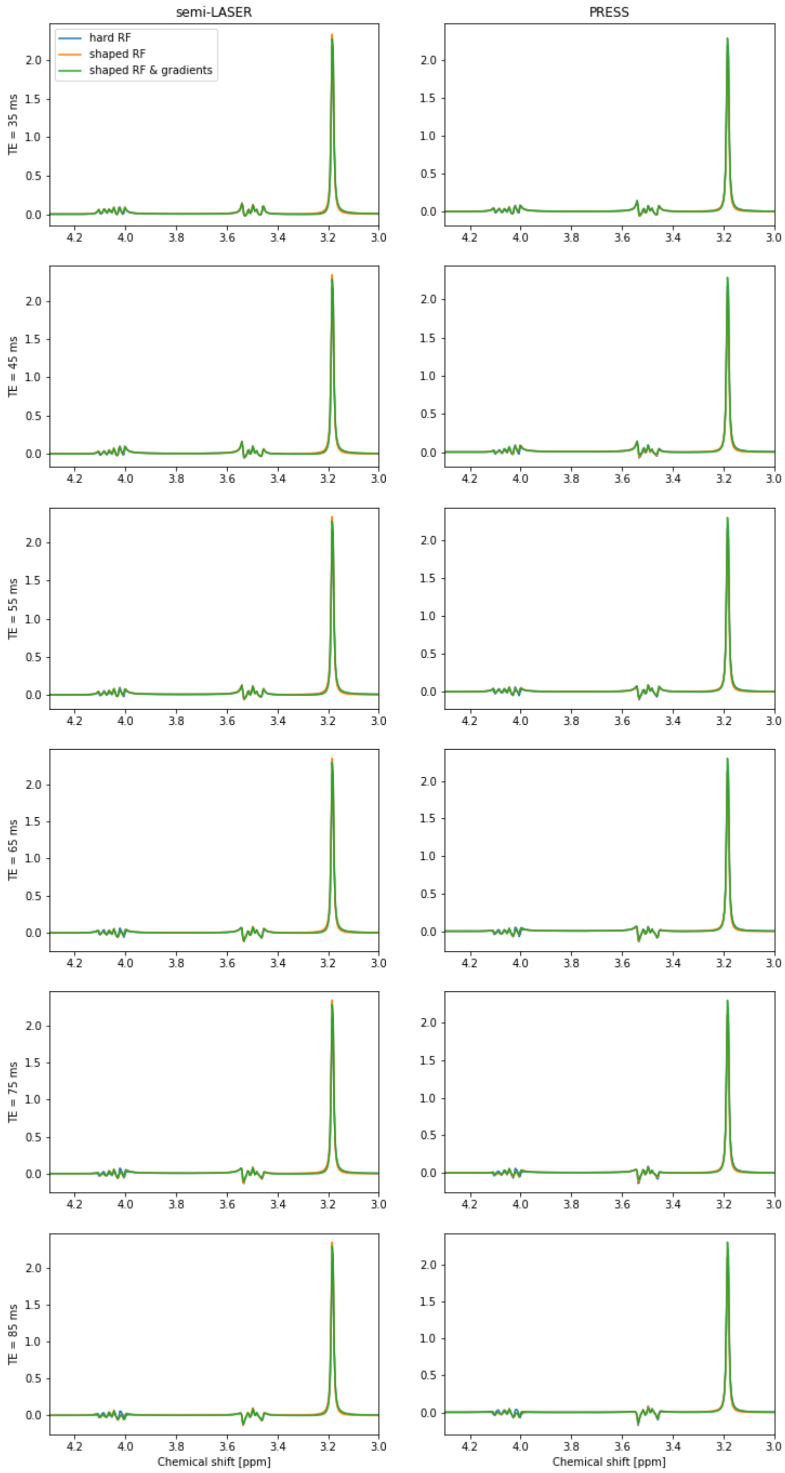

semi-LASER
PRESS
hard RF
shaped RF
shaped RF & gradients
TE = 35 ms
TE = 45 ms
TE = 55 ms
TE = 65 ms
TE = 75 ms
TE = 85 ms
Chemical shift [ppm]
Chemical shift [ppm]

S4. Creatine

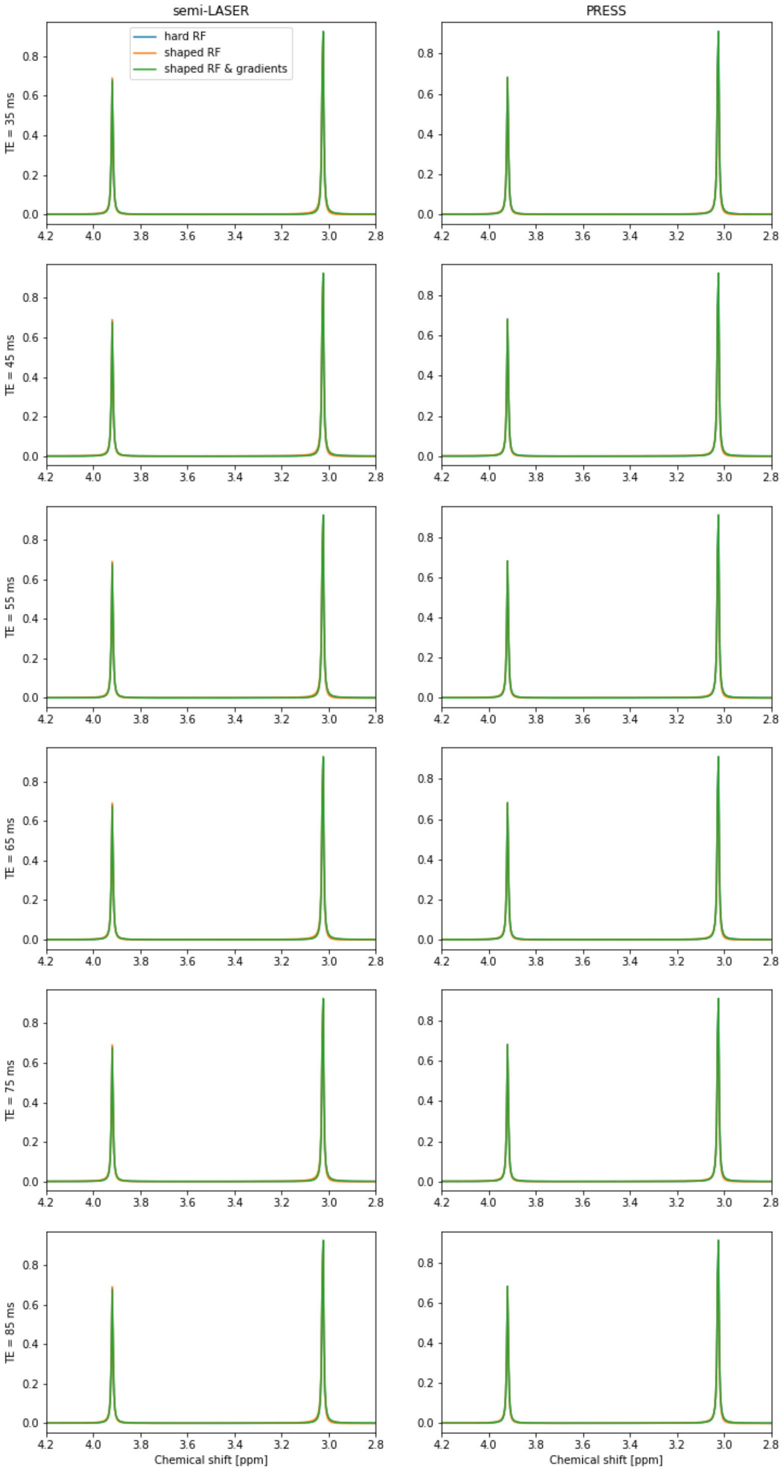

semi-LASER
PRESS
hard RF
shaped RF
shaped RF & gradients
TE = 35 ms
TE = 45 ms
TE = 55 ms
TE = 65 ms
TE = 75 ms
TE = 85 ms
Chemical shift [ppm]

S5. Glutamate

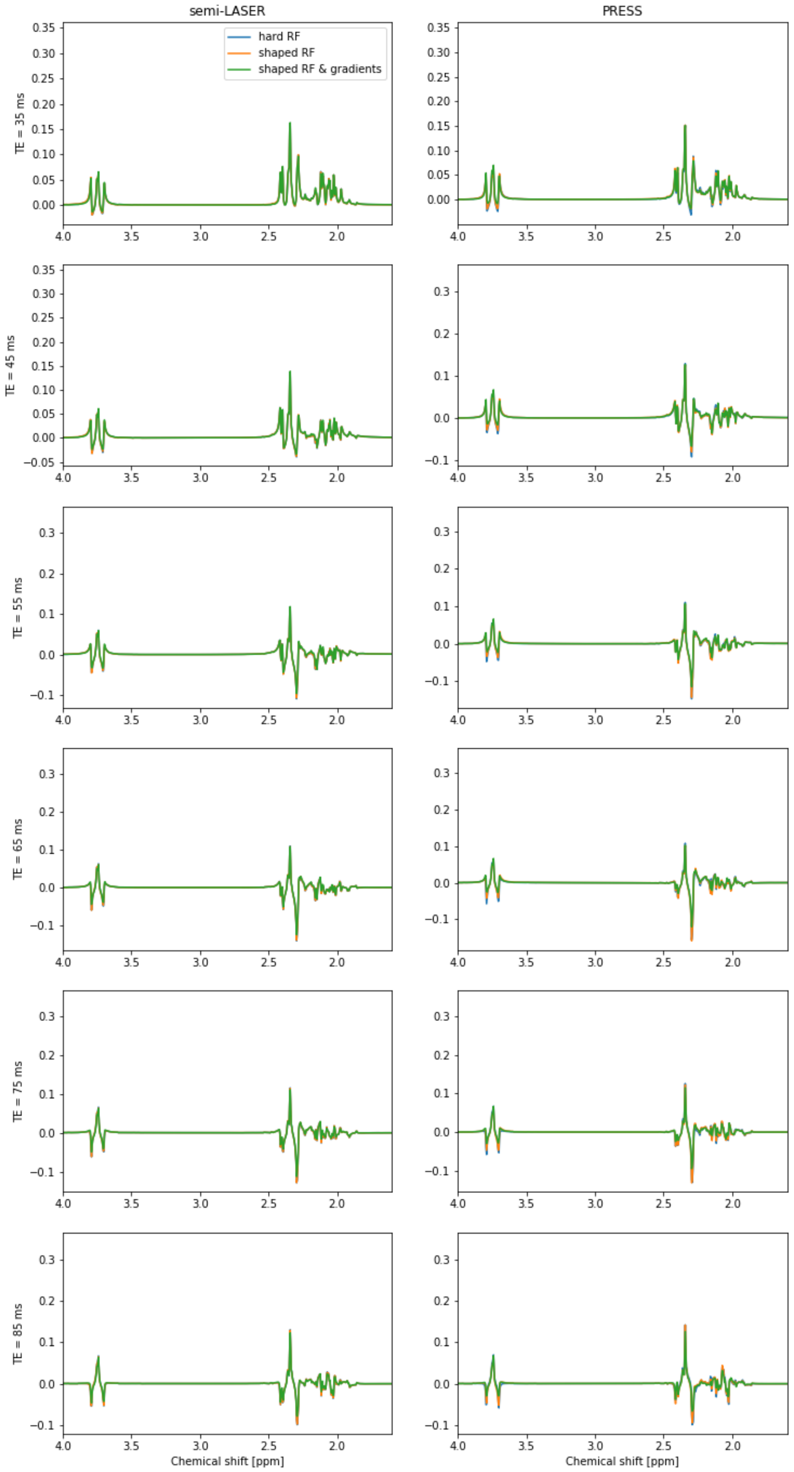

S6. Myo-Inositol

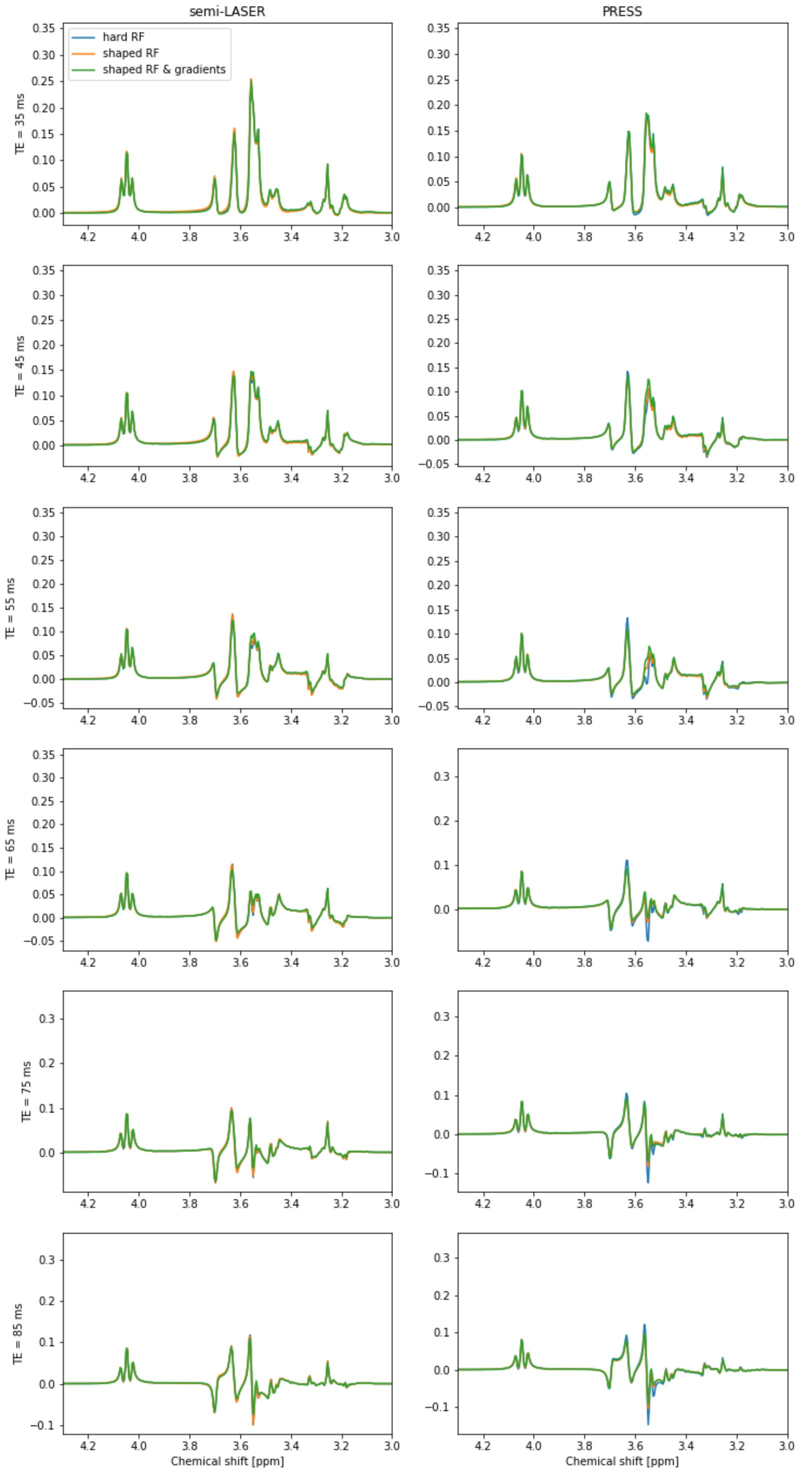

S7. NAA

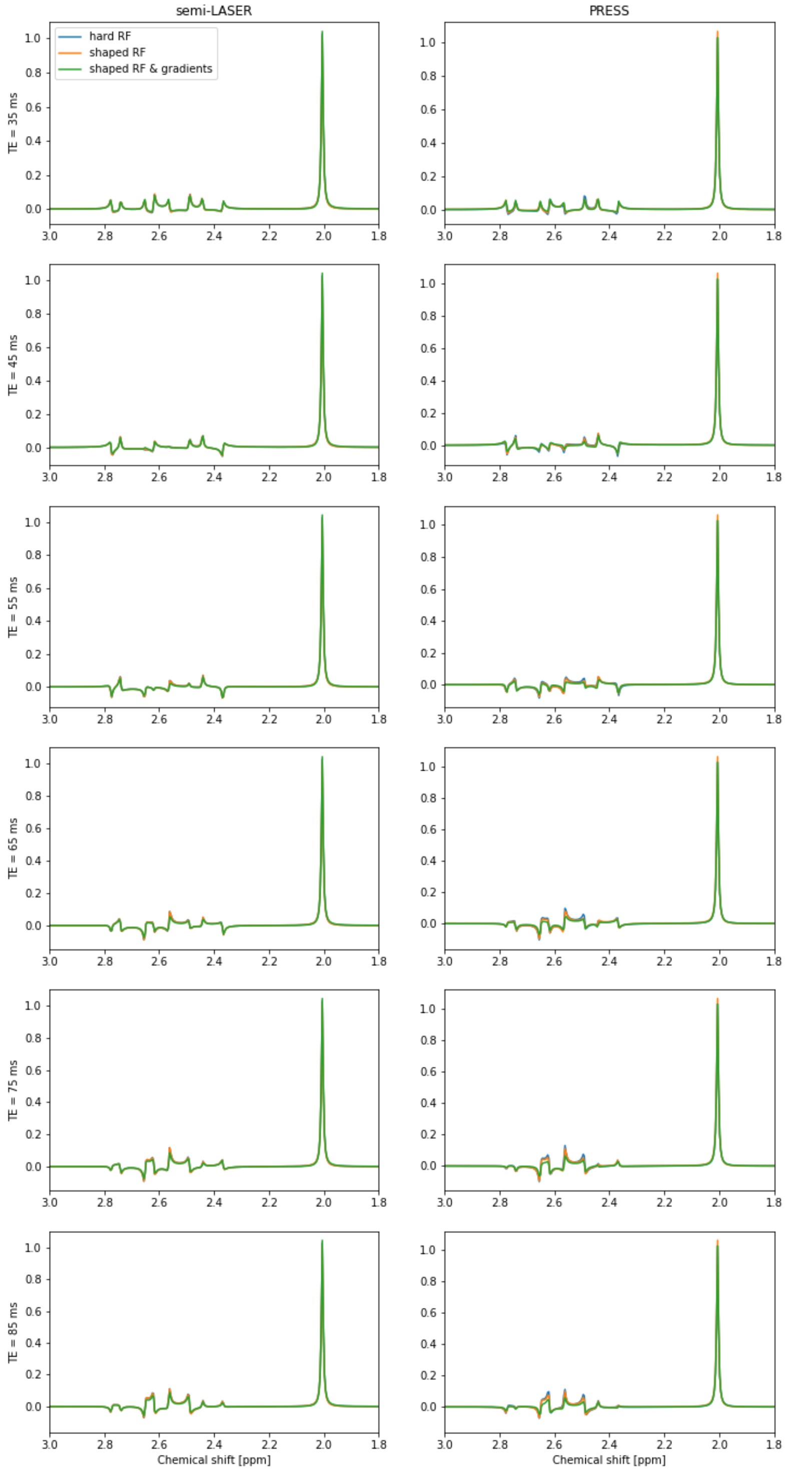

semi-LASER
PRESS
hard RF
shaped RF
shaped RF & gradients
TE = 35 ms
TE = 45 ms
TE = 55 ms
TE = 65 ms
TE = 75 ms
TE = 85 ms
Chemical shift [ppm]
Chemical shift [ppm]

Supplementary figures S8-S12. Measured phantom spectra (black), LCModel fit (colored) and residual (black elevated) for each simulation method and pulse sequence.

S8. TE = 45 ms

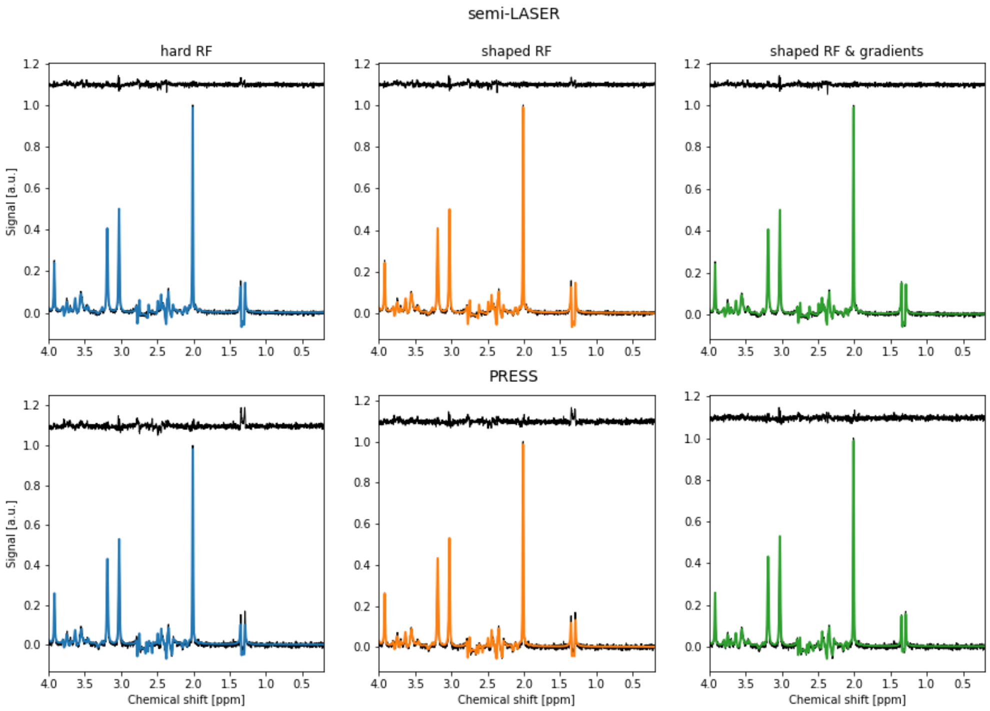

S9. TE = 55 ms

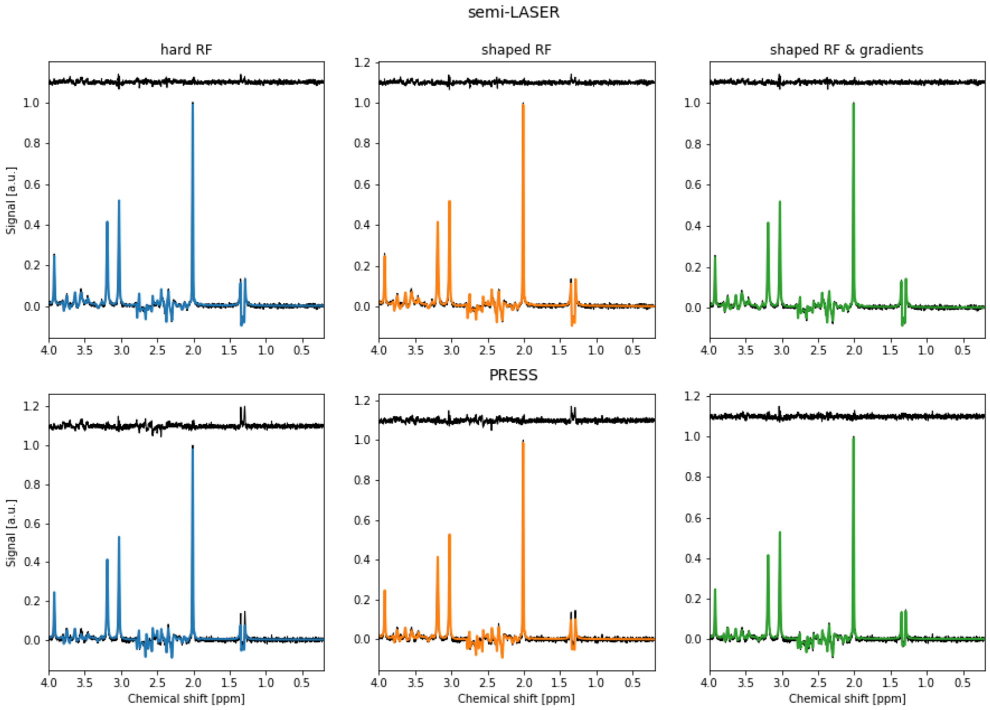

S10. TE = 65 ms

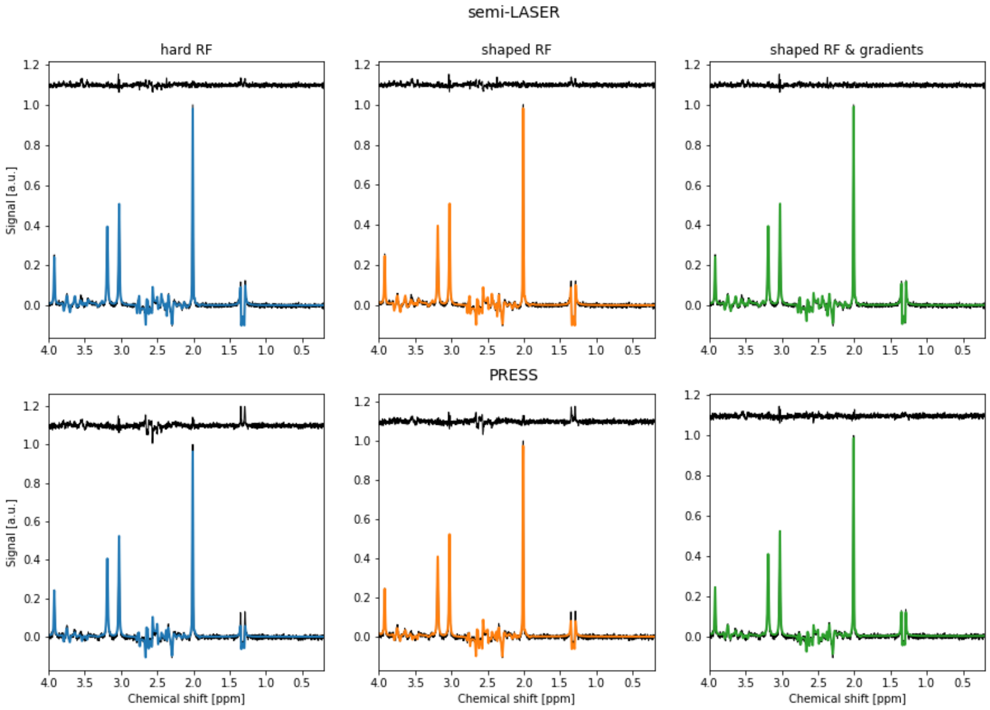

S11. TE = 75 ms

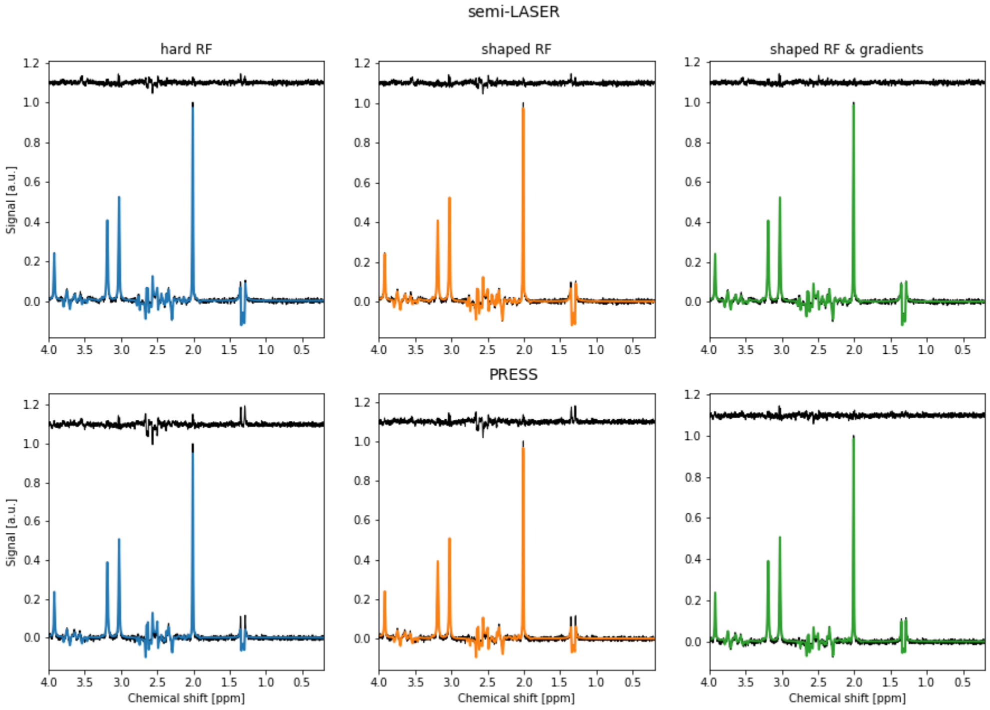

<u>S12. TE = 85 ms</u>

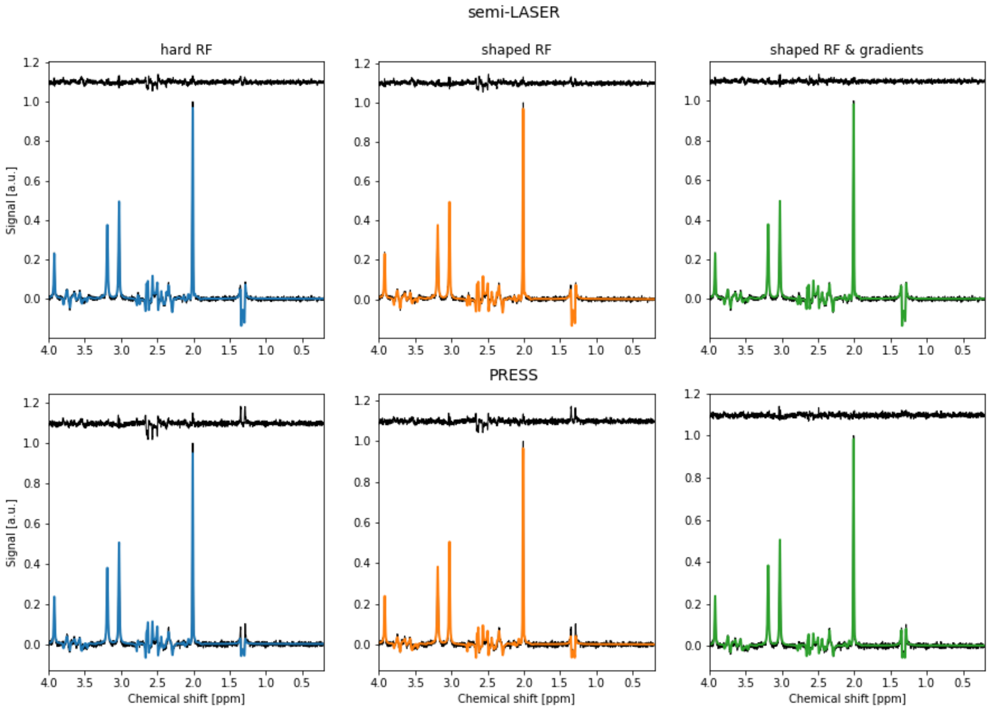

Figure S13. Estimated phantom metabolite concentrations corrected for T2 relaxation (extrapolated to TE = 0 ms based on monoexponential fit of multi-TE data) and bias relative to specified concentrations

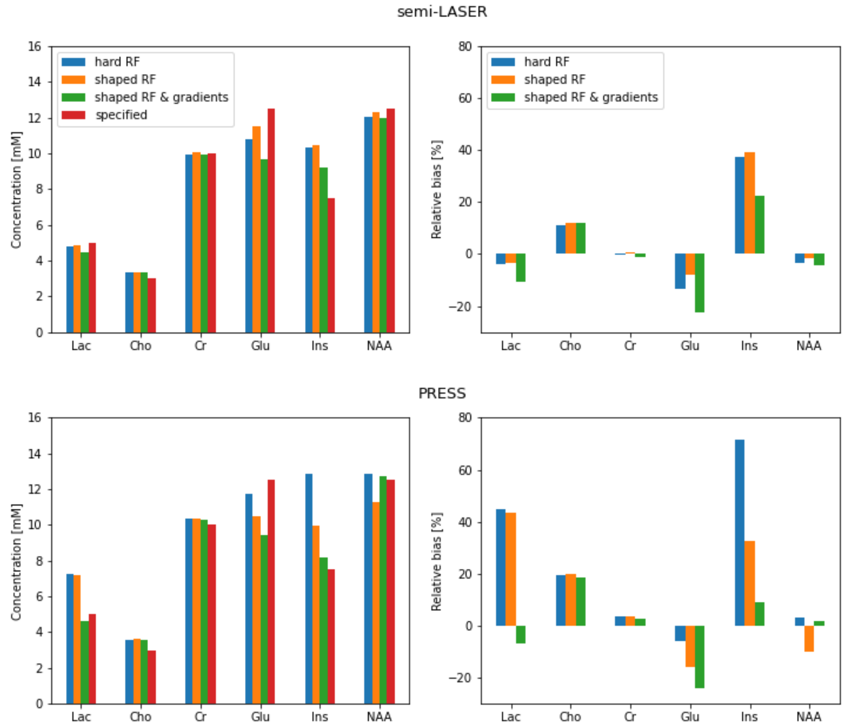

Figure S14. Estimated phantom metabolite concentrations vs TE and exponential model fit (related to Fig. S13)

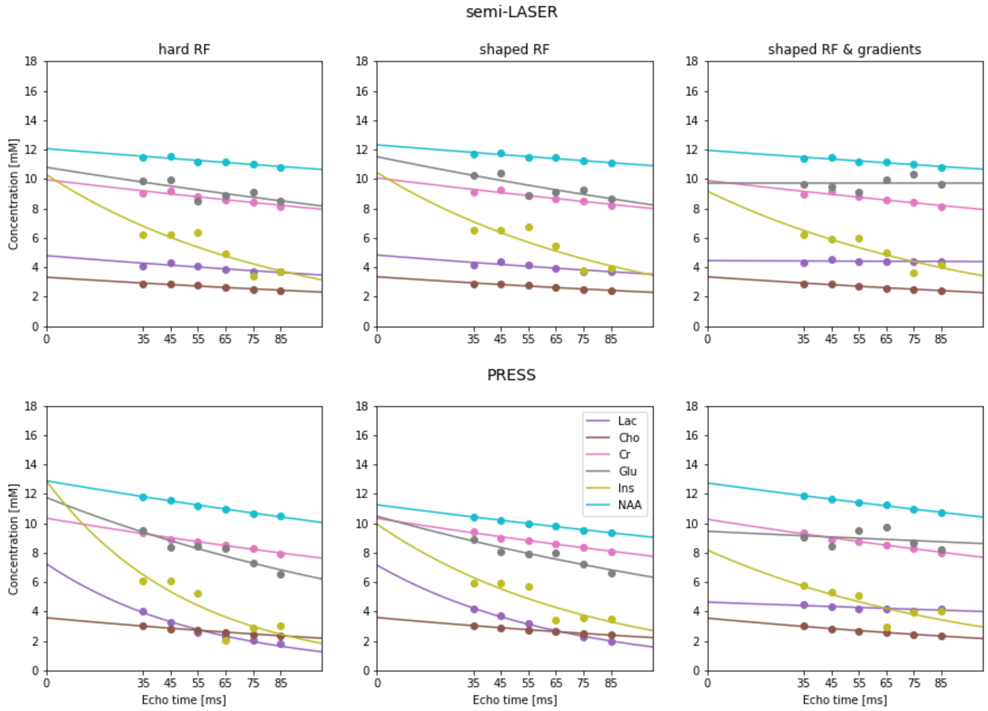

Supplementary figures S15-S19. Measured *in vivo* spectra (black), LCModel fit (colored) and residual (black elevated) for each simulation method and pulse sequence.

S15. TE = 45 ms

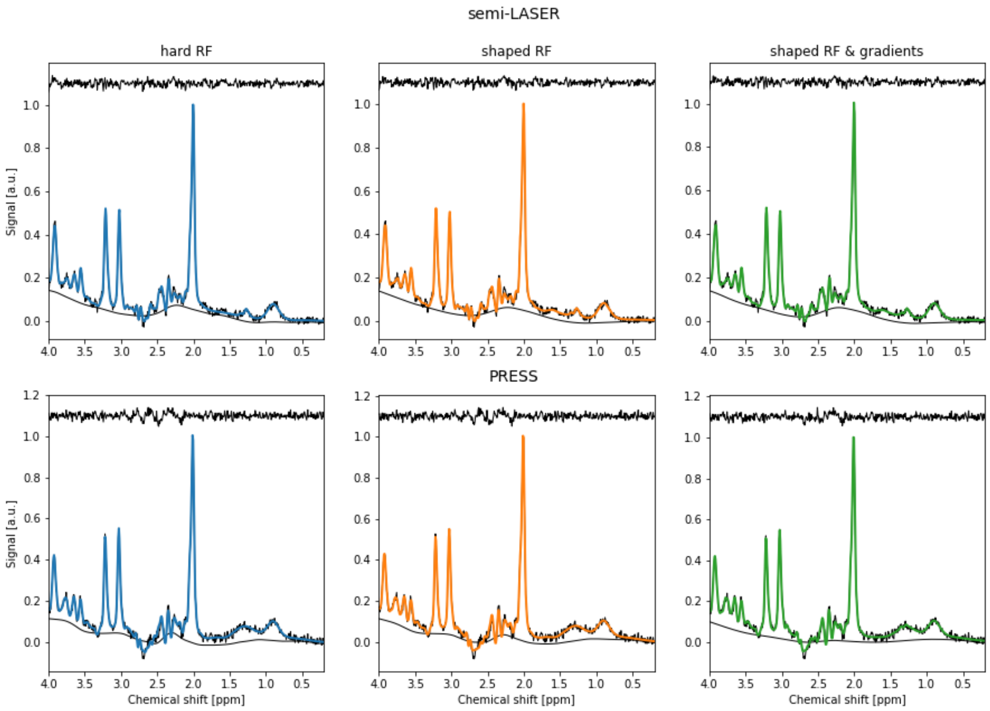

S16. TE = 55 ms

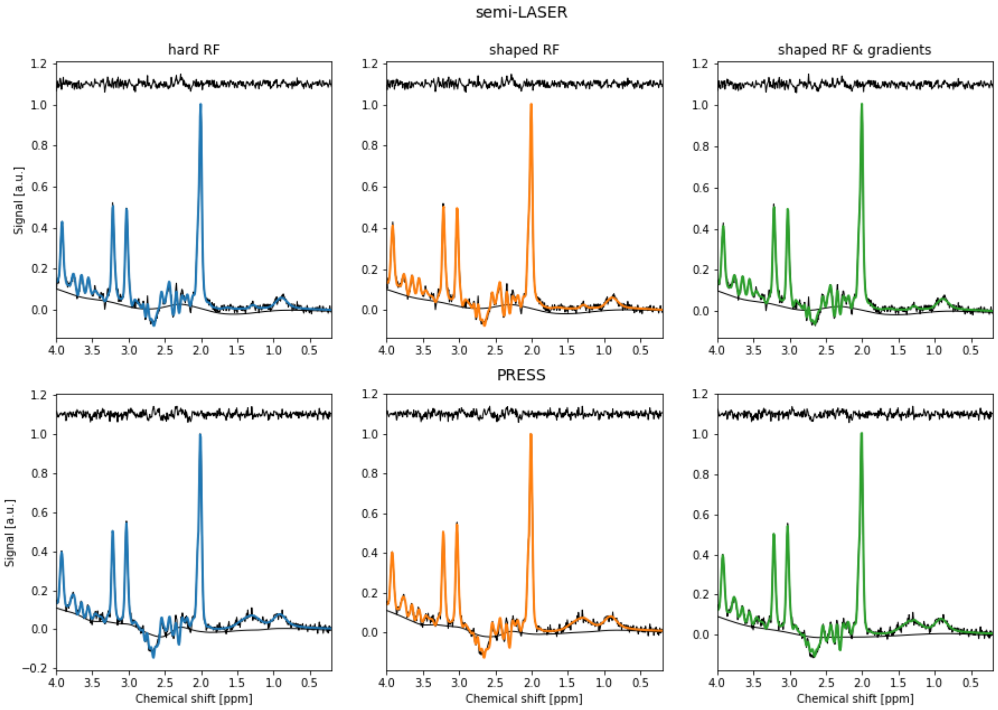

S17. TE = 65 ms

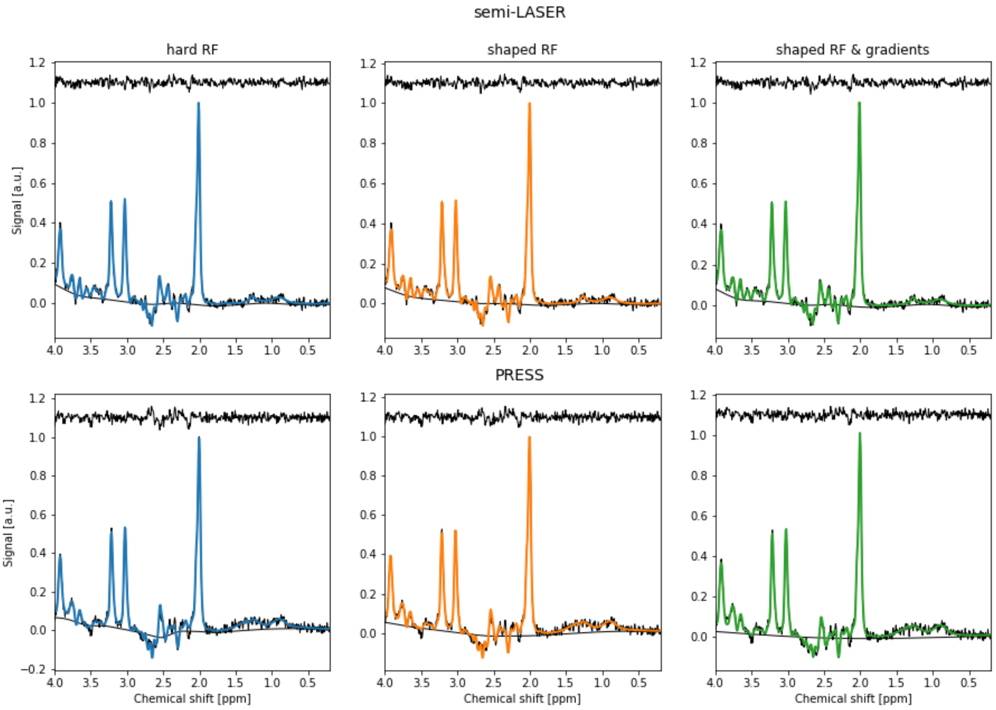

S18. TE = 75 ms

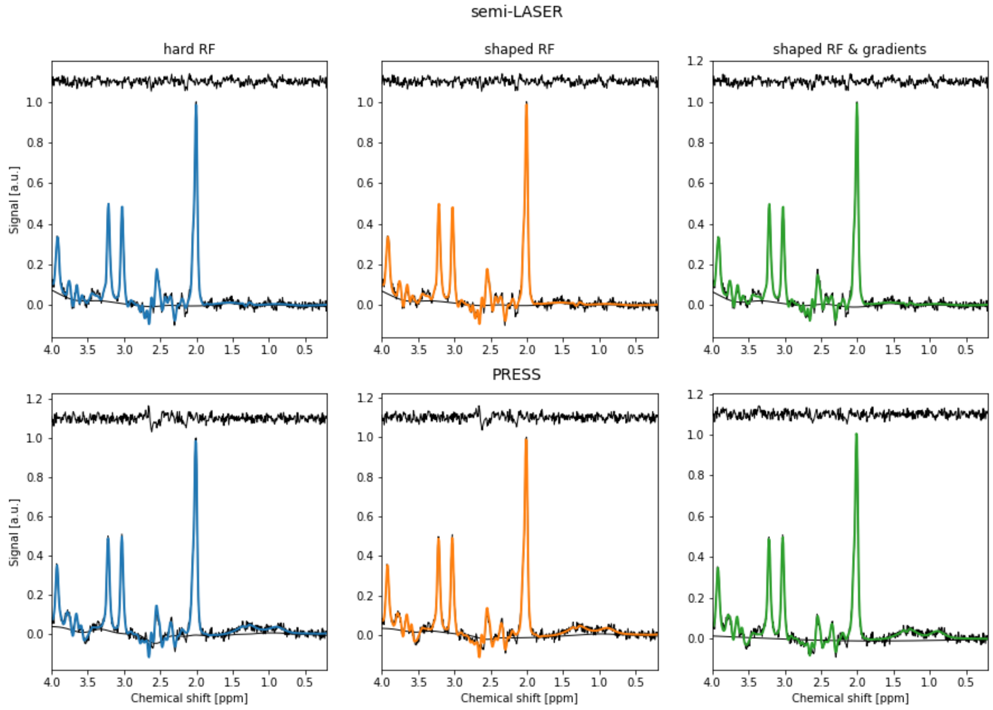

S19. TE = 85 ms

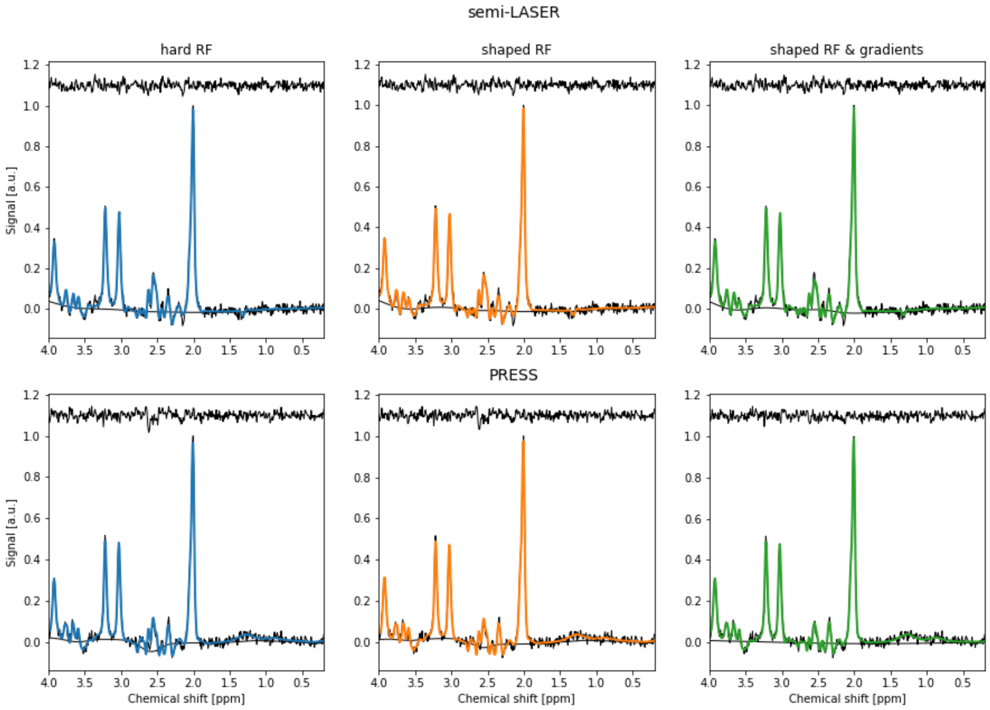

semi-LASER
hard RF
shaped RF
shaped RF & gradients
PRESS
Signal [a.u.]
Chemical shift [ppm]

Fig S20 Estimated in vivo metabolite concentrations corrected for T2 relaxation (extrapolated to TE = 0 ms based on monoexponential fit of multi-TE data)

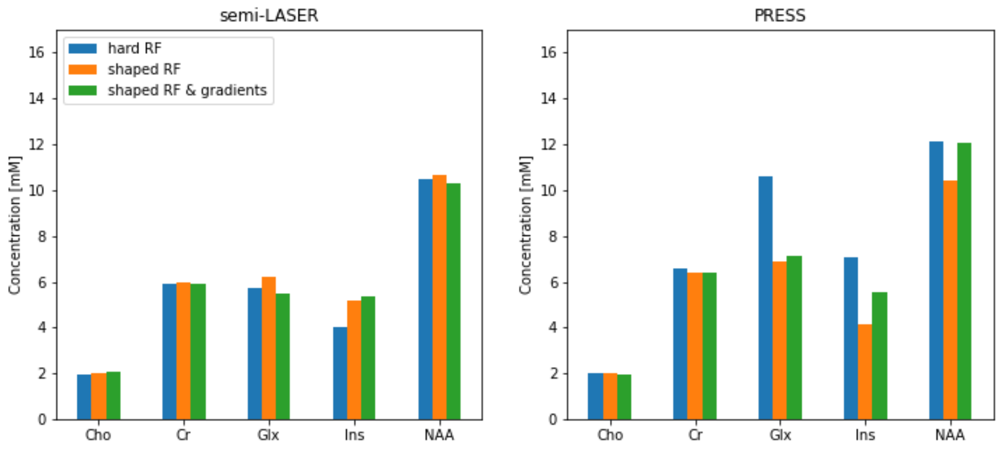

Supplementary figures S21. Estimated *in vivo* metabolite concentrations vs TE and exponential model fit (related to Fig. S20)

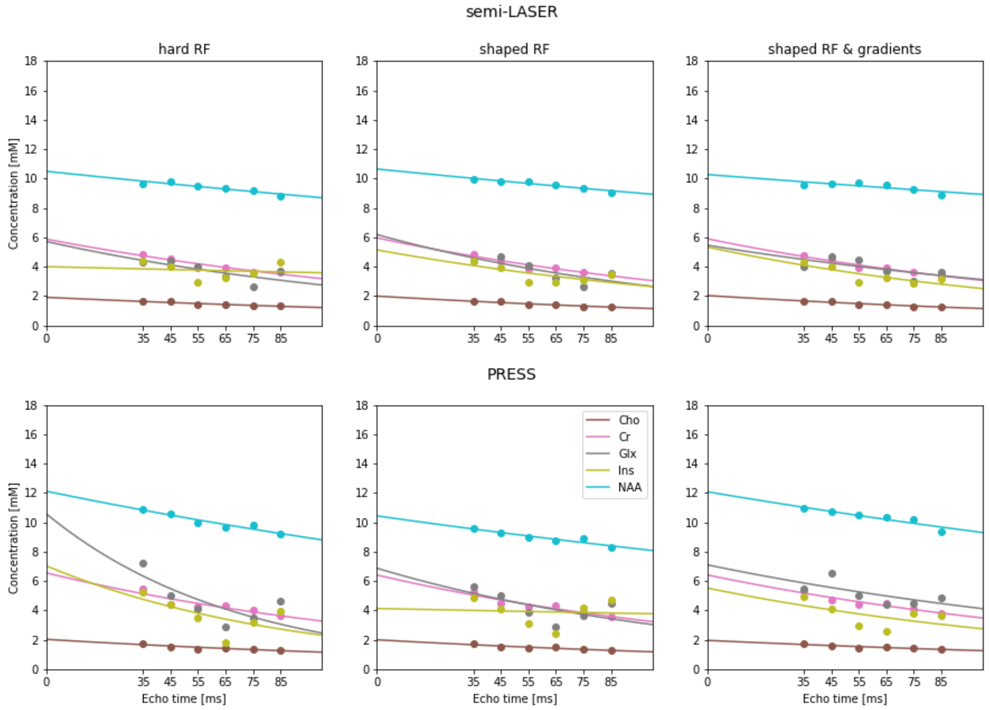

Supplement: Supplementary file 1 — Supplementary file1 Supplementary figures: Fig S1 VOI position used for in vivo measurements. Figs S2-S7 as fig 1 but for all metabolites and TEs. Figs S8-S12 as fig 3 but for remaining TEs. Fig S13 Estimated phantom metabolite concentrations corrected for T2 relaxation (extrapolated to TE = 0 ms based on monoexponential fit of multi-TE data) and bias relative to specified concentrations. Fig S14 estimated phantom metabolite concentrations vs TE and exponential model fit (related to Fig 6). Figs S15-S19 as Fig 5 but for remaining TEs. Fig S20 Estimated in vivo metabolite concentrations corrected for T2 relaxation (extrapolated to TE = 0 ms based on monoexponential fit of multi-TE data). Fig S21 Estimated in vivo metabolite concentrations vs TE and exponential model fit (related to Supporting Fig S20) (PDF 2032 KB) [file 10334_2020_900_MOESM1_ESM.pdf]
